# Mepolizumab reduces systemic corticosteroid-related toxicities and healthcare burden in patients with eosinophilic granulomatosis with polyangiitis and hypereosinophilic syndrome: a retrospective database study

**DOI:** 10.3389/falgy.2026.1843149

**Published:** 2026-08-12

**Authors:** Thanai Pongdee, Jared Silver, Arijita Deb, Francois Laliberte, Chi Gao, Annalise Hilts, Kaixin Zhang, Daisy Liu, Amy G. Edgecomb

**Affiliations:** 1 Division of Allergic Diseases, Mayo Clinic, Rochester, MN, United States; 2 Clinical Development, Amgen, Thousand Oaks, CA, United States; 3 US Real-World Evidence and Health Outcomes Research, Respiratory, GSK, Collegeville, PA, United States; 4 Groupe d’analyse, Ltée, Montreal, QC, Canada; 5 Analysis Group, Inc., Boston, MA, United States; 6 US Real-World Evidence and Health Outcomes Research, Anti-infectives and Respiratory, GSK, Collegeville, PA, United States

**Keywords:** biologic, eosinophilic granulomatosis with polyangiitis, healthcare resource utilization, hypereosinophilic syndrome, real-world evidence, systemic corticosteroid

## Abstract

**Background:**

Systemic corticosteroids (SCS) remain the cornerstone of eosinophilic granulomatosis with polyangiitis (EGPA) and hypereosinophilic syndrome (HES) therapy but are associated with substantial toxicity. Mepolizumab, an anti-interleukin-5 biologic, has demonstrated steroid-sparing effects in addition to EGPA and HES disease control, yet evidence of reductions in SCS-related complications is limited.

**Methods:**

This retrospective cohort study used US claims data from the Komodo Research Database to quantify SCS-related complications, healthcare resource utilization, and costs in patients with EGPA or HES treated with mepolizumab vs. chronic SCS. Patients were stratified as mepolizumab (300 mg) or chronic SCS users (≥6 months continuous SCS use with >7.5 mg/day for EGPA and >10 mg/day for HES) based on treatment received during a 6-month landmark period post-index (first claim of mepolizumab or SCS). An inverse probability of treatment weighting approach was applied to the cohorts to minimize confounding. Outcomes were assessed up to 12 months post-landmark.

**Results:**

578 patients with EGPA (305 mepolizumab users vs. 273 chronic SCS users) and 272 patients with HES (161 vs. 111) were included. In patients with EGPA, rates of any SCS-related complications were 39% lower with mepolizumab compared with chronic SCS (rate ratio [95% confidence interval]: 0.61 [0.49, 0.76], *p* < 0.001). Mepolizumab users had 57% lower rate of all-cause hospitalizations (0.43 [0.27, 0.69], *p* < 0.001), and lower outpatient and emergency department (ED) visits vs. chronic SCS. Patients with HES had significantly lower rates of any SCS-related complications with mepolizumab vs. chronic SCS (0.59 [0.42, 0.82], *p* = 0.002). Mepolizumab users had 66% lower rates of all-cause hospitalizations (0.34 [0.17, 0.65], *p* = 0.001), and lower outpatient visits relative to chronic SCS users. In EGPA and HES SCS-complication-related hospitalizations and ED visits were lower with mepolizumab compared with chronic SCS. SCS-complication-related medical costs per patient per year were $16,194 (*p* = 0.008) and $3,976 (*p* = 0.676) lower, in the mepolizumab vs. chronic SCS cohort of patients with EGPA and HES, respectively.

**Conclusion:**

This first of its kind study suggests that real-world mepolizumab use is associated with reduced SCS-related complications in EGPA and HES, reinforcing its clinical and economic steroid-sparing benefits in reducing healthcare burden among EGPA and HES populations.

## 1 Introduction

Eosinophilic granulomatosis with polyangiitis (EGPA) and hypereosinophilic syndrome (HES) are rare eosinophil-driven diseases characterized by eosinophilic tissue infiltration, leading to inflammation and multi-organ dysfunction (1–3). The multi-systemic manifestations of both diseases contribute to the significant disease burden experienced by patients and drive high healthcare resource utilization (HRU) including frequent hospitalizations and visits to the emergency department (ED) (4–7).

While the treatment landscape for EGPA and HES continues to evolve, systemic corticosteroids (SCS) remain the cornerstone of care (8–13). Given the chronic nature of the diseases, patients frequently require prolonged courses of SCS leading to dependence, and often relapse during SCS tapering (8, 11, 14). Despite their widespread use, the use of SCS is associated with acute and chronic complications, with documented dose-dependent and cumulative toxicity (12, 15–17). These include acute complications such as infections and gastrointestinal side effects (i.e., gastric ulcers and dyspepsia) (18), and chronic conditions including osteoporosis, hypertension, sleep disturbances, and type 2 diabetes (17). In a prospective study of glucocorticoid toxicity among patients with antineutrophil cytoplasmic antibody-associated vasculitis (AAV; including EGPA), 92% of patients had SCS-associated complications (19).

Treatment goals for EGPA and HES include induction and maintenance of remission, and reducing relapses (8, 20). For remission induction in patients with EGPA or HES, high-dose SCS (starting at 30–60 mg/day) are generally administered, often in combination with additional immunosuppressants in severe disease (8, 12). For remission maintenance in patients with EGPA, treatment guidelines advocate for use of steroid-sparing agents and tapering SCS to the lowest possible effective dose with the aim of reducing SCS-related morbidity, including the use of more targeted therapies such as biologics (8–11). The use of steroid-sparing agents in patients with HES is also recommended to minimize steroid-related adverse effects (13, 20, 21).

Mepolizumab is a first-in-class humanized monoclonal antibody that specifically targets interleukin-5 (IL-5). By binding to IL-5, mepolizumab treatment reduces the proliferation, activation and survival of eosinophils, restoring eosinophils to healthy physiological levels (22, 23). Mepolizumab is approved for the treatment of severe asthma with an eosinophilic phenotype, EGPA, HES, chronic rhinosinusitis with nasal polyps and chronic obstructive pulmonary disease in multiple regions worldwide (24–26). For HES and EGPA, the approved dose is mepolizumab 300 mg administered subcutaneously, every 4 weeks (24, 26). For patients with EGPA, the pivotal Phase III MIRRA trial demonstrated that with mepolizumab more patients achieved remission and spent more time in remission, had fewer relapses, and demonstrated steroid-sparing benefits (22, 27). Steroid-sparing benefits were further evidenced in open-label and real-world studies, where significant reductions in the average daily dose of SCS were observed following the initiation of mepolizumab (28–30). In patients with HES, steroid-sparing benefits of mepolizumab have also been demonstrated in a clinical trial, an open-label extension, and in real-world studies (31–33). Although it is hypothesized that steroid reductions with mepolizumab translate to meaningful improvement in SCS-related complications for patients with EGPA and HES, this has not yet been demonstrated.

To address this gap, we conducted an administrative claims study to quantify SCS-related adverse events and associated HRU and costs among patients with EGPA and HES, and compared outcomes in patients receiving mepolizumab therapy vs. chronic SCS use.

## 2 Materials and methods

### 2.1 Study design and participant eligibility

**Figure 1:**
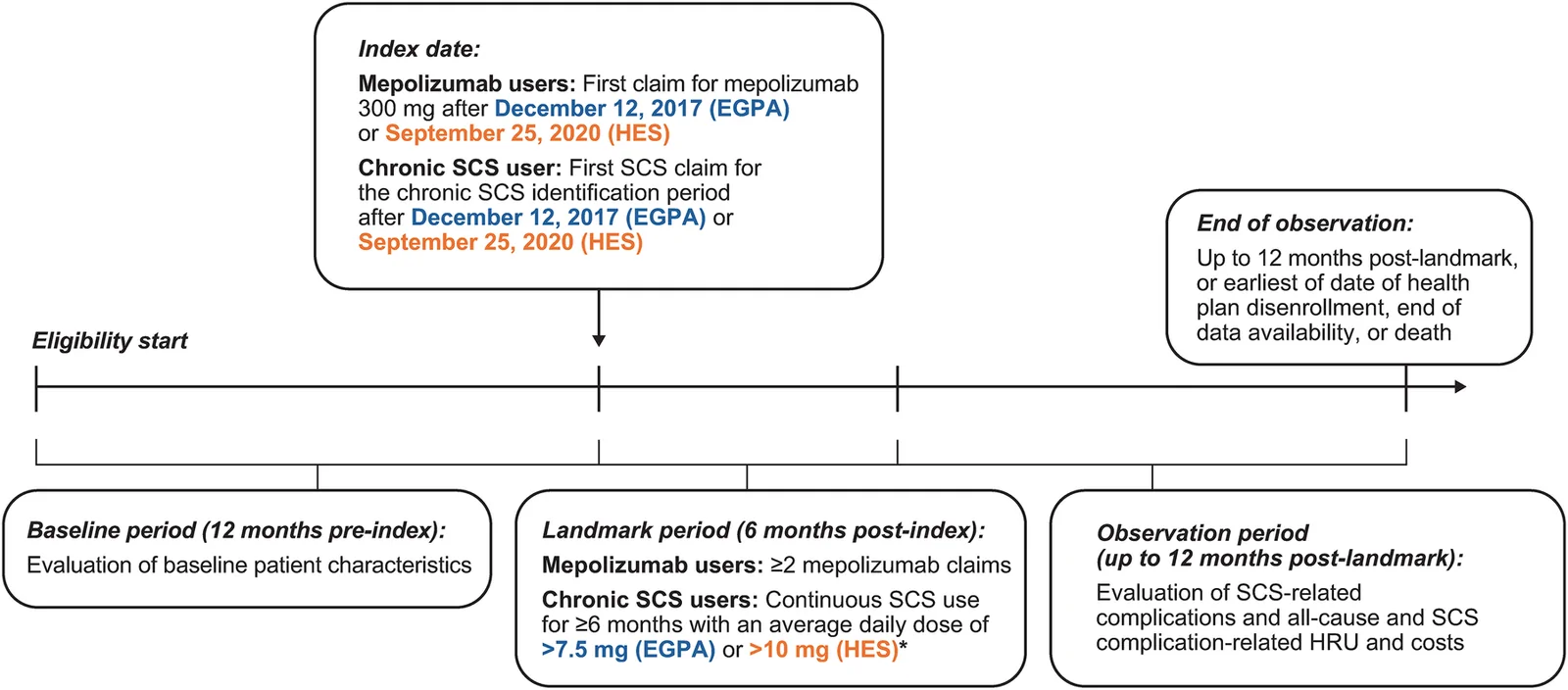
EGPA and HES study design. *Continuous use of oral or parenteral SCS with an average daily dose equivalent of prednisone. EGPA, eosinophilic granulomatosis with polyangiitis; HES, hypereosinophilic syndrome; HRU, healthcare resource utilization; SCS, systemic corticosteroid.

This was a retrospective, longitudinal cohort study (GSK ID: 221046) using medical and pharmacy claims data from the Komodo Research Database from January 1, 2016, to December 31, 2023, comparing mepolizumab users with chronic SCS users in patients with EGPA or HES. The Komodo Research database includes de-identified data from more than 320 million patients enrolled in a healthcare plan in the US. Patients were identified as mepolizumab users or chronic SCS users based on treatment received during the 6-month landmark period after their first claim for mepolizumab or, separately, their first claim for SCS after December 12, 2017 (EGPA index date) or after September 25, 2020 (HES index date). The landmark period served as the treatment effect period. The observation period was defined as up to 12 months from the end of the landmark period to the date of health plan disenrollment, end of data availability, or death (whichever occurred first), and allowed for comparison of outcomes (Figure 1).

Identification of patients with EGPA is shown in Supplementary Figure S1. For patients with EGPA, mepolizumab users had ≥2 medical or pharmacy claims for mepolizumab 300 mg within 6 months of the index date; chronic SCS users had ≥6 months of continuous use of oral or parenteral SCS with an average daily dose equivalent to prednisone >7.5 mg from the index date. Eligible patients with EGPA were also required to have continuous coverage for ≥12 months before the index date (baseline period) and ≥6 months after the index date (landmark period), to be ≥18 years of age at the index date, and to have ≥1 diagnosis of EGPA (International Classification of Diseases, 10th Revision, Clinical Modification [ICD-10-CM: M30.1]) during the baseline period. Key exclusion criteria included any patient with ≥1 medical or pharmacy claim for mepolizumab 300 mg prior to index date for mepolizumab users, and any patient with ≥1 medical or pharmacy claim for mepolizumab during the eligibility period (i.e., the period of eligible insurance coverage that included the entire study period of baseline, landmark, or observation) for chronic SCS users. Patients treated with omalizumab, reslizumab, benralizumab, dupilumab, or tezepelumab during the eligibility period were excluded for both mepolizumab users and chronic SCS users.

Identification of patients with HES is shown in Supplementary Figure S2. For patients with HES, mepolizumab users had ≥2 medical or pharmacy claims for mepolizumab 300 mg within 6 months of the index date. Chronic SCS users had ≥6 months of continuous use of oral or parenteral SCS with an average daily dose equivalent to prednisone >10 mg from index date. Eligible patients were also required to have ≥12 months continuous coverage during the baseline period, ≥6 months continuous coverage during the landmark period, to be ≥12 years of age at index, and to have ≥1 diagnosis of HES (ICD-10-CM: D72.11) during the baseline period. Key exclusion criteria for mepolizumab users and chronic SCS users with HES are the same as in patients with EGPA.

### 2.2 Study objectives and outcomes

The primary objective was to evaluate and compare the rate of any SCS-related complications among patients with EGPA or HES treated with mepolizumab 300 mg subcutaneously vs. chronic SCS during the observation period. Secondary objectives included comparing the rate of any acute and any chronic SCS-related complications, and evaluating all-cause and SCS-related HRU and healthcare costs among patients with EGPA or HES treated with mepolizumab vs. chronic SCS. Patients with EGPA and patients with HES were evaluated as two independent study populations.

Any SCS-related complications were defined as any acute (e.g., gastrointestinal, infections, bone and muscle-related, and cardiovascular identified in an inpatient or ED setting only) or chronic (e.g., bone- and muscle-related, cardiovascular, hematologic, metabolic and endocrine, ophthalmologic, and central nervous system) SCS-related complications, listed in Supplementary Table S1 (18, 34, 35), and identified using primary or secondary ICD-10-CM diagnosis codes.

HRU measures included hospitalizations, ED visits, and outpatient visits (including and excluding outpatient visits for mepolizumab administration). Healthcare costs included medical costs related to hospitalization, ED visits, outpatient visits, as well as pharmacy costs. Healthcare costs were reported including and excluding cost of mepolizumab and mepolizumab administration. Outpatient visits were classified as mepolizumab administration visits if the medical claim contained a Healthcare Common Procedure Coding System (HCPCS) code for mepolizumab or if the medical claim had a procedure code for subcutaneous or intramuscular drug administration within the earlier of the following two criteria—28 days on or after a pharmacy claim for mepolizumab, or on or before the next medical claim with a mepolizumab-administration-specific HCPCS code.

### 2.3 Statistical analysis

Patient demographics and clinical characteristics were assessed during the 12-month baseline period. These were described using descriptive statistics, including mean, standard deviation (SD), and median for continuous variables, and frequencies and proportions for categorical variables, and were compared using standardized differences.

An inverse probability of treatment weighting (IPTW) approach based on the propensity score (PS) for being treated with mepolizumab was applied to balance measured baseline characteristics between the mepolizumab and chronic SCS cohorts, minimizing potential confounding from patient characteristics associated with treatment assignment. The PS, representing each patient's conditional probability of receiving mepolizumab based on observable covariates, was used to derive weights that create a pseudo-population in which the distribution of covariates in the population is independent of treatment assignment. The covariates were selected based on clinical relevance, standardized difference (> 20%) and covariate prevalence (> 10%), as listed in Supplementary Methods (Propensity score covariates) (36). Thus, the PS with IPTW approach attempted to mimic the balance achieved in a randomized trial and allowed for the estimation of an average treatment effect with reduced confounding from measured variables. Covariate balance was assessed post-weighting using standardized differences, with a threshold of 20% used to indicate meaningful imbalance. After IPTW application, the majority of covariates achieved standardized differences well below 20% in both the EGPA and HES populations (Table 1), indicating that the cohorts were generally well balanced across measured baseline characteristics (36). All results presented in this study are based on IPTW-weighted cohorts, with IPTW-weighted rate ratios or differences.

**Table 1:** Demographics and clinical characteristics for patients with EGPA and HES (IPTW weighted cohorts).

Characteristic	EGPA			HES		
	Mepolizumab cohort	Chronic SCS cohort	Std. diff. (%)	Mepolizumab cohort	Chronic SCS cohort	Std. diff. (%)
	*N* = 305	*N* = 273		*N* = 161	*N* = 111	
Observation period length^†^						
Days, mean (SD) [median]	306.0 (105.0) [365.0]	316.0 (98.1) [365.0]	9.90	282.4 (121.2) [365.0]	258.3 (121.9) [340.0]	19.90
Age at index date						
Years, mean (SD) [median]	54.9 (15.2) [56.7]	55.5 (15.3) [58.1]	3.90	53.7 (17.5) [57.2]	55.1 (16.9) [57.3]	7.80
Sex, *n* (%)						
Female	179 (58.8)	143 (52.6)	12.70	87 (54.0)	56 (50.0)	7.90
Male	124 (40.7)	122 (44.8)	8.30	74 (46.0)	55 (50.0)	7.90
Unknown	1 (0.5)	7 (2.7)	17.60	-	-	-
US geographic region, *n* (%)						
Northeast	97 (31.7)	86 (31.4)	0.80	58 (36.3)	40 (35.8)	1.00
South	70 (22.8)	59 (21.5)	3.20	34 (21.2)	22 (20.0)	2.90
Midwest	73 (24.0)	77 (28.0)	9.10	42 (25.9)	32 (28.6)	6.20
West	65 (21.4)	52 (19.1)	5.70	27 (16.7)	17 (15.6)	3.10
Insurance plan type^†^, *n* (%)						
Commercial	203 (66.6)	187 (68.3)	3.70	96 (59.4)	65 (59.0)	0.80
Medicare	72 (23.5)	71 (26.0)	5.80	47 (29.4)	33 (29.6)	0.50
Medicaid	30 (9.9)	15 (5.6)	15.80	18 (11.2)	13 (11.4)	0.50
Quan-CCI^‡^						
Mean (SD) [median]	2.1 (1.8) [1.0]	2.1 (1.8) [1.0]	2.40	2.2 (1.9) [2.0]	2.3 (2.3) [2.0]	3.10
Select baseline immunosuppressants, *n* > 10, *n* (%)						
Any use	148 (48.6)	128 (47.0)	3.20	38 (23.7)	26 (23.7)	0.10
Azathioprine	55 (18.0)	43 (15.7)	5.90	3 (1.7)	0 (0.4)	12.40
Methotrexate	49 (16.1)	32 (11.8)	12.50	10 (6.4)	4 (3.5)	13.10
Mycophenolate	20 (6.6)	15 (5.7)	3.70	4 (2.2)	1 (1.1)	8.50
Rituximab	22 (7.2)	37 (13.4)	20.60^a^	0 (0.0)	2 (1.8)	18.90
Hydroxychloroquine	12 (3.8)	12 (4.5)	3.50	8 (5.1)	4 (3.4)	8.40
Cyclophosphamide	3 (1.0)	12 (4.3)	20.20^a^	1 (0.8)	0 (0.0)	12.30
Hydroxyurea	4 (1.3)	1 (0.2)	12.90	11 (6.6)	7 (6.7)	0.40
Cumulative SCS dosage during baseline^§^						
mg, mean (SD) [median]	1,843.2 (1,511.2) [1,590.0]	1,846.7 (1,443.4) [1,620.0]	0.20	1,360.8 (1,223.7) [1,100.0]	1,392.2 (1,459.2) [825.0]	2.30
EGPA relapses^¶^, mean (SD) [median]						
Any EGPA relapses	2.3 (2.5) [1.0]	2.2 (2.6) [1.0]	3.70	-	-	-
Major EGPA-related relapses**	0.2 (0.7) [0.0]	0.2 (0.5) [0.0]	5.50	-	-	-
HES flares^††^						
Mean (SD) [median]	–	–	–	1.9 (2.1) [1.0]	2.2 (2.1) [2.0]	15.60
Asthma diagnosis during eligibility period^‡‡^						
n (%)	259 (84.8)	228 (83.7)	2.90	109 (67.6)	76 (68.3)	1.5
Number of asthma-related exacerbations^§§^ during baseline, mean (SD) [median]						
IP exacerbations	0.1 (0.5) [0.0]	0.1 (0.5) [0.0]	5.00	0.1 (0.4) [0.0]	0.1 (0.5) [0.0]	3.60
OP/ED exacerbations	0.8 (1.3) [0.0]	1.1 (2.2) [0.0]	14.80	0.4 (0.9) [0.0]	0.4 (0.8) [0.0]	4.80

* Denotes Std. diff. > 20%, used to suggest meaningful imbalance between cohort;.

† evaluated at the index date.

‡ CCI algorithm was based on coding algorithms for defining comorbidities in ICD-9-CM and ICD-10 administrative data (51).

§ cumulative dose was defined as the sum of the quantity × prednisone equivalent strength over the given time period in milligrams. For medical claims with missing quantity information, quantity was imputed as 1.

¶ EGPA relapses were identified using diagnoses of EGPA, vasculitis, and other conditions; high-dose SCS; and initiation of rituximab/cyclophosphamide, after excluding visits associated with mepolizumab administration. Relapses were required to have 30 days of relapse-free time beforehand.

** major EGPA relapses were identified as the subgroup of EGPA relapses occurring in the IP setting.

†† HES flares were identified as an increase in prednisone equivalent OCS dosage of >10 mg/day for at least 5 days OR ≥1 pharmacy or medical claim for any newly initiated immunosuppressive HES therapy. The definition of HES flares in this context is based on clinical trial (23).

‡‡ refers to asthma-associated diagnosis codes captured from start of continuous eligibility to the end of continuous eligibility, i.e., the period of eligible insurance coverage encompassing the baseline, landmark, and observation, patients may have asthma codes coded prior to eligibility period which have not been captured.

§§ IP exacerbations were defined as an asthma-related IP visit. OP/ED exacerbations were defined as an asthma-related OP visit or asthma-related ED visit with at least one claim for an SCS (i.e., intramuscular, intravenous, or oral) within −4/+5 days of the encounter.

ED, emergency department; EGPA, eosinophilic granulomatosis with polyangiitis; HES, hypereosinophilic syndrome; HRU, health resource utilization; ICD-10-CM, International Classification of Diseases, 10th Revision, Clinical Modification; IP, inpatient; IPTW, inverse probability of treatment weighting; OCS, oral corticosteroid, OP, outpatient; Quan-CCI, Quan-Charlson Comorbidity Index; SCS, systemic corticosteroid; SD, standard deviation; Std. diff, standardized difference; US, United States.

For all cohorts, the rate of SCS-related complications and HRU were assessed during the observation period, and were calculated as number of events per patient-year (PPY). The relative rate of SCS-related complications and HRU between weighted treatment arms was analyzed using generalized linear models with a Poisson distribution (i.e., Poisson regression) or a negative binomial distribution to account for observed overdispersion, respectively, with 95% confidence intervals (CIs) and *p*-values using robust standard errors.

Healthcare costs were reported per person per year (PPPY) and were assessed during the observation period. All costs were inflation-adjusted to 2023 US dollars (USD) based on the US Medical Care Consumer Price Index from the US Department of Labor Bureau of Labor Statistics. Healthcare costs were analyzed using logistic regression to estimate the marginal probability of non-zero costs, and a generalized linear model with a gamma distribution and log-link function was used to estimate the predicted cost differences conditioned on the probability of non-zero costs. 95% CIs and *p*-values were generated using non-parametric bootstrap procedures.

All analyses were conducted using SAS Enterprise Guide software Version 7.15 (SAS Institute Inc., Cary, NC).

### 2.4 Sensitivity analyses

Two sensitivity analyses (dosage and diagnosis) were performed to test the robustness of study results, as described in Supplementary Methods (Sensitivity analysis methods).

## 3 Results

### 3.1 Demographics and clinical characteristics

A total of 8,543 patients with EGPA, and 11,241 patients with HES were identified in the Komodo Research Database in the period from January 1, 2016, to December 31, 2023. Following application of all study eligibility criteria, 578 patients with EGPA were included in the study, stratified as 305 patients treated with mepolizumab and 273 treated with chronic SCS (Supplementary Figure S1).

Of the 272 patients with HES included in the study, 161 patients were treated with mepolizumab and 111 received chronic SCS (Supplementary Figure S2).

The mean (SD) observation period for patients with EGPA included in the study was 306.0 (105.0) days for patients treated with mepolizumab, and 316.0 (98.1) days for patients treated with chronic SCS. In patients with HES, the mean (SD) observation period was 282.4 (121.2) days for mepolizumab users, and 258.3 (121.9) for chronic SCS users.

For both patients with EGPA and HES, demographics are reported for IPTW-weighted cohorts and were well-balanced between the mepolizumab and chronic SCS cohorts post IPTW-weighting (Table 1). Cumulative SCS dose, reported as prednisone equivalent mg, over the baseline period was similar between mepolizumab and chronic SCS cohorts (mean [SD]), for both patients with EGPA (1,843.2 [1,511.2] vs. 1,846.7 [1,443.4] mg) and HES (1,360.8 [1,223.7] vs. 1,392.2 [1,459.2] mg). Additional baseline clinical characteristics, including baseline medication use, EGPA relapses and HES flares, and asthma exacerbations were similar between cohorts (Table 1). Baseline all-cause HRU and healthcare costs were also balanced between treatment cohorts in patients with EGPA and HES (Supplementary Table S2).

>Baseline frequencies of acute and chronic SCS-related complications were also similar between the mepolizumab and chronic SCS cohorts (mean [SD]), for patients with EGPA (acute: 1.8 [6.1] vs. 2.5 [9.4] PPY; chronic: 11.7 [16.0] vs. 13.1 [18.5] PPY) and HES (acute: 2.1 [3.9] vs. 2.7 [5.0] PPY; chronic: 13.0 [18.2] vs. 13.9 [23.3] PPY). The most common baseline Elixhauser comorbidities for both patients with EGPA and HES in the mepolizumab and chronic SCS cohorts were hypertension, rheumatoid arthritis/collagen vascular disease (excluding EGPA in patients already diagnosed with EGPA), and cardiac arrhythmias (Table 2).

**Table 2:** Baseline Elixhauser comorbidities and SCS-related complications in patients with EGPA and HES (IPTW weighted cohorts).

EGPA	Mepolizumab cohort *N* = 305	Chronic SCS cohort *N* = 273	Std. diff (%)
Most common Elixhauser comorbidities, *n* (%)			
Hypertension	140 (45.9)	117 (43.0)	5.70
Rheumatoid arthritis/collagen vascular disease	113 (36.9)	100 (36.5)	1.00
Cardiac arrhythmias	79 (26.0)	68 (24.9)	2.60
Obesity	78 (25.6)	54 (19.7)	14.10
Hypothyroidism	55 (18.0)	61 (22.2)	10.40
Fluid and electrolyte disorders	57 (18.6)	55 (20.2)	3.90
Diabetes	51 (16.7)	52 (19.0)	6.00
Congestive heart failure	40 (13.2)	41 (15.2)	5.60
Valvular disease	41 (13.4)	40 (14.5)	3.30
Liver disease	38 (12.3)	38 (14.0)	5.00
Number of SCS-related complications during 12-month baseline period, mean (SD)			
Any acute SCS-related complications	1.8 (6.1)	2.5 (9.4)	8.40
Any chronic SCS-related complications	11.7 (16.0)	13.1 (18.5)	8.00

HES	Mepolizumab cohort *N* = 161	Chronic SCS cohort *N* = 111	Std. diff (%)
Most common Elixhauser comorbidities, *n* (%)			
Hypertension	79 (48.8)	51 (45.5)	6.60
Cardiac arrhythmias	63 (38.8)	49 (43.8)	10.00
Rheumatoid arthritis/collagen vascular disease	64 (40.0)	34 (30.4)	20.00
Valvular disease	41 (25.5)	23 (20.9)	10.90
Deficiency anemias	31 (19.1)	28 (24.8)	13.80
Obesity	36 (22.5)	25 (22.3)	0.50
Diabetes	32 (20.0)	20 (18.4)	4.10
Congestive heart failure	37 (22.8)	25 (22.7)	0.30
Liver disease	22 (13.8)	16 (14.0)	0.50
Peripheral vascular disorders	31 (19.4)	12 (10.9)	23.70^a^
Number of baseline SCS-related complications during 12-month baseline period, mean (SD)			
Any acute SCS-related complications	2.1 (3.9)	2.7 (5.0)	13.70
Any chronic SCS-related complications	13.0 (18.2)	13.9 (23.3)	4.40

a Denotes Std. diff. >20%, which was used to suggest meaningful imbalance between cohorts.

EGPA, eosinophilic granulomatosis with polyangiitis; HES, hypereosinophilic syndrome; IPTW, inverse probability of treatment weighting; PPY, per patient-year; SCS, systemic corticosteroid; SD, standard deviation; Std. diff., standardized difference.

### 3.2 SCS-related complications in patients with EGPA

During the observation period, patients with EGPA in the mepolizumab cohort had an observed rate of 9.56 any SCS-related complications PPY compared with 18.09 PPY in the chronic SCS cohort. This translated to a significant 39% lower rate of any SCS-related complication in mepolizumab users compared with chronic SCS users (rate ratio [95% CI]: 0.61 [0.49, 0.76], *p* < 0.001).

**Figure 2:**
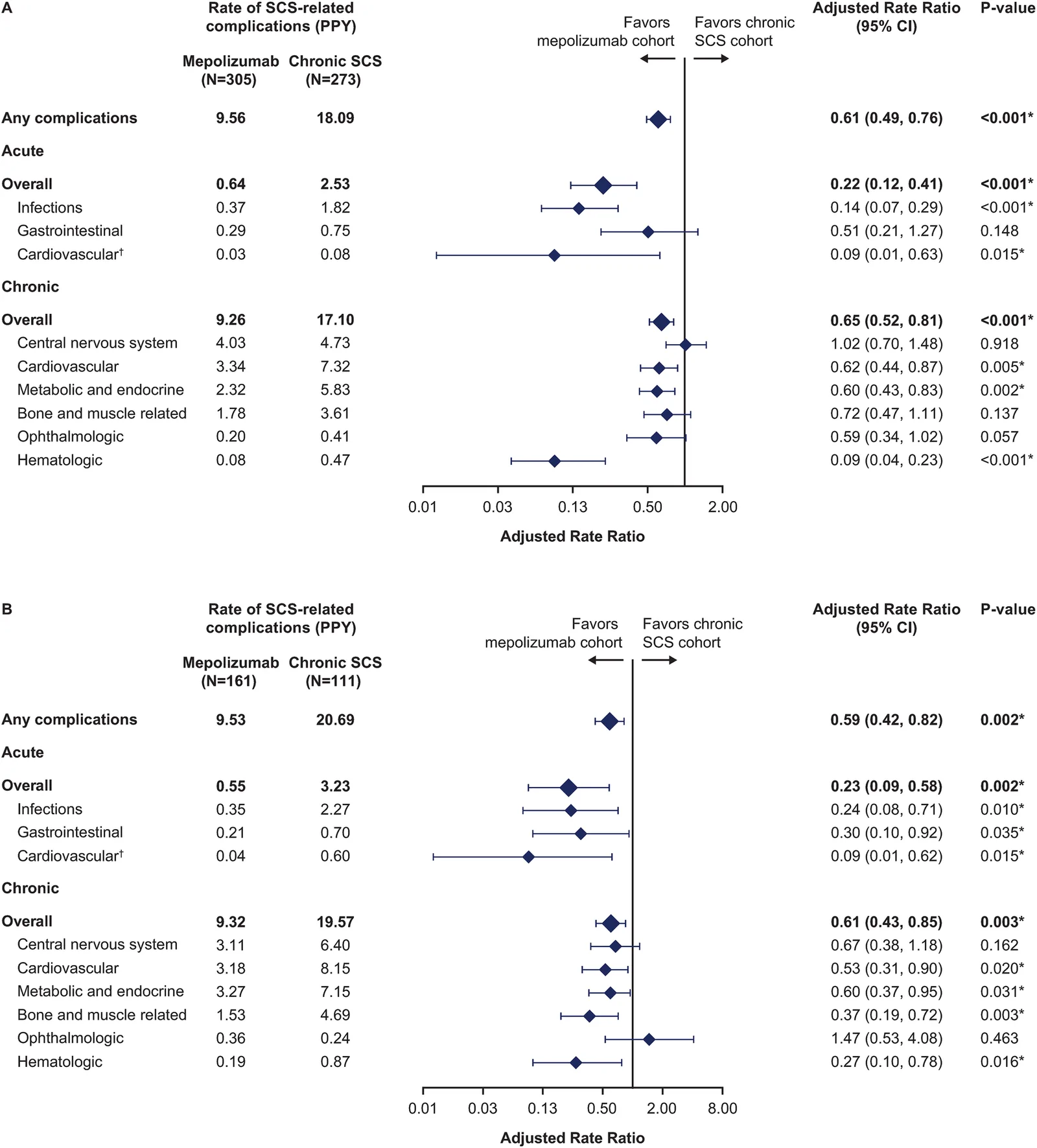
SCS-related complications among mepolizumab vs. chronic SCS cohorts for **(A)** EGPA, **(B)** HES. *p < 0.05 adjusted rate ratio achieving statistical significance; ^†^myocardial infarction was the only acute SCS-related cardiovascular complication. CI, confidence interval; EGPA, eosinophilic granulomatosis with polyangiitis; HES, hypereosinophilic syndrome; PPY, per patient-year; SCS, systemic corticosteroid.

Mepolizumab users also had a 78% lower rate of any acute complications compared with chronic SCS users (0.22 [0.12, 0.41], *p* < 0.001), with significantly lower rates of infections, and cardiovascular complications (myocardial infarction)*.* For chronic complications, mepolizumab use was associated with a 35% lower rate overall compared with chronic SCS use (0.65 [0.52, 0.81], *p* < 0.001), including significantly lower rates of cardiovascular, metabolic and endocrine, and hematologic chronic complications (Figure 2A).

### 3.3 SCS-related complications in patients with HES

Patients with HES treated with mepolizumab had an observed rate of 9.53 SCS-related complications PPY compared with a rate of 20.69 PPY in patients treated with chronic SCS during the observation period. This translated to a significant 41% lower rate of any SCS-related complications in mepolizumab users compared with chronic SCS users (rate ratio [95% CI]: 0.59 [0.42, 0.82], *p* = 0.002).

Mepolizumab users also had a 77% lower rate of acute complications compared with chronic SCS users (0.23 [0.09 0.58], *p* = 0.002), with significantly lower rates of infections, gastrointestinal symptoms, and cardiovascular complications (myocardial infarction). For chronic complications, mepolizumab use was associated with a 39% lower rate overall compared with chronic SCS use (0.61 [0.43, 0.85], *p* = 0.003), including significantly lower rates of cardiovascular, metabolic and endocrine, bone- and muscle-related, and hematologic complications (Figure 2B).

### 3.4 SCS usage in patients with EGPA and HES

Descriptive data on SCS use in patients with EGPA and HES is reported in Supplementary Table S3, including cumulative SCS dosage, in milligrams, number of SCS dispensings, and mean daily dose between mepolizumab and chronic SCS users.

### 3.5 Healthcare resource utilization

**Figure 3:**
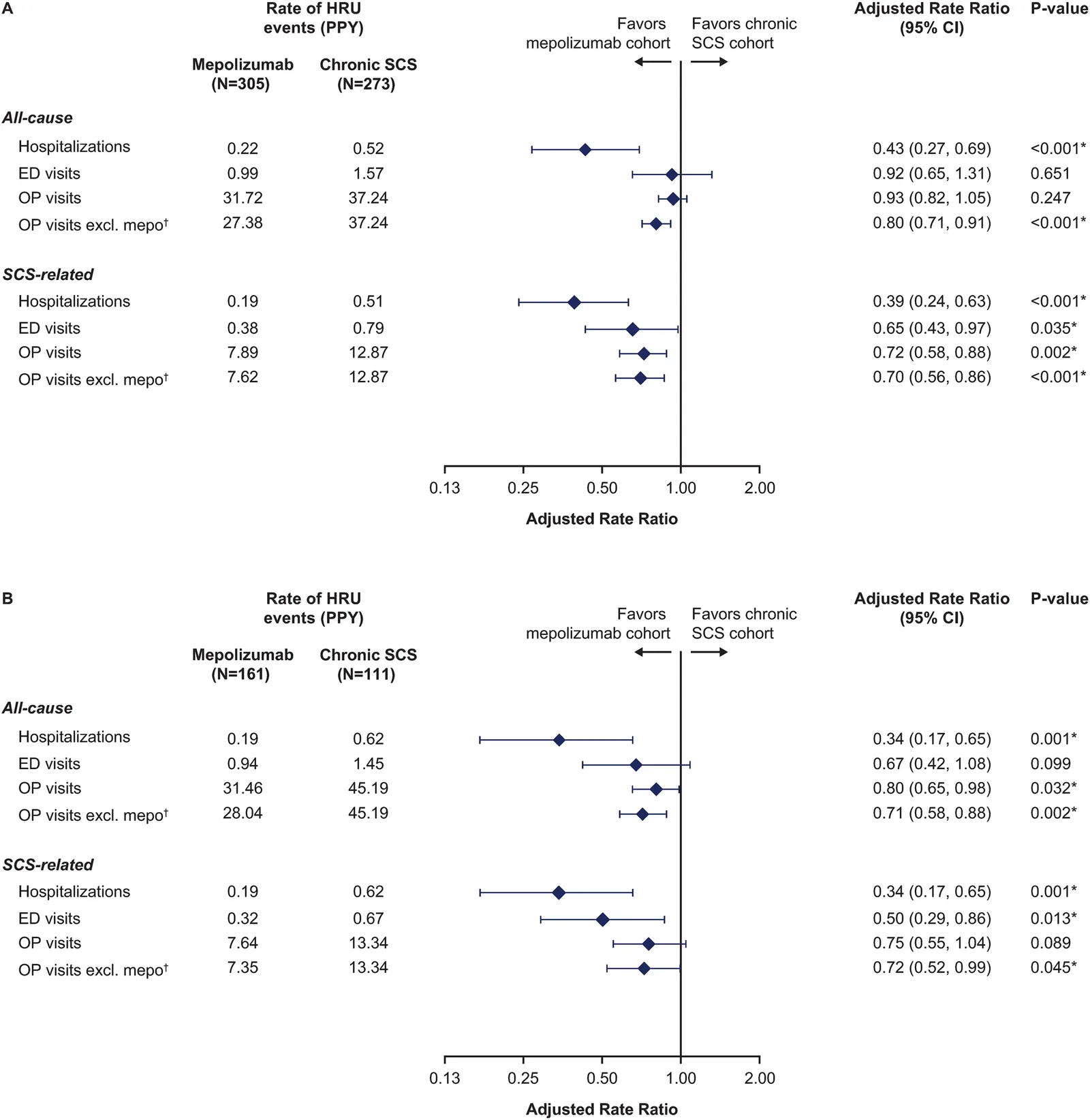
HRU among mepolizumab vs. chronic SCS cohorts for **(A)** EGPA and **(B)** HES. *p < 0.05, adjusted rate ratio achieving statistical significance; ^†^OP visits associated with mepolizumab administration were excluded. CI, confidence interval; EGPA, eosinophilic granulomatosis with polyangiitis; ED, emergency department; HES, hypereosinophilic syndrome; HRU, healthcare resource utilization; OP, outpatient; PPY, per patient-year; SCS, systemic corticosteroid.

Patients with EGPA treated with mepolizumab had a significant 57% (*p* < 0.001) lower rate of all-cause hospitalizations compared with the chronic SCS cohort, as well as a 20% (*p* < 0.001) lower rate of non-mepolizumab administration-related outpatient visits. SCS complication-related HRU was also significantly lower among mepolizumab users compared with chronic SCS users (Figure 3A).

Patients with HES treated with mepolizumab had a significant 66% (*p* = 0.001) lower rate of all-cause hospitalizations compared with the chronic SCS cohort, as well as a 20% (*p* = 0.032) lower rate of non-mepolizumab administration-related outpatient visits. Regarding SCS complication-related HRU, patients treated with mepolizumab had lower rates of hospitalizations, and ED visits compared with the chronic SCS cohort (Figure 3B).

### 3.6 All-cause and SCS-complication-related healthcare costs

**Figure 4:**
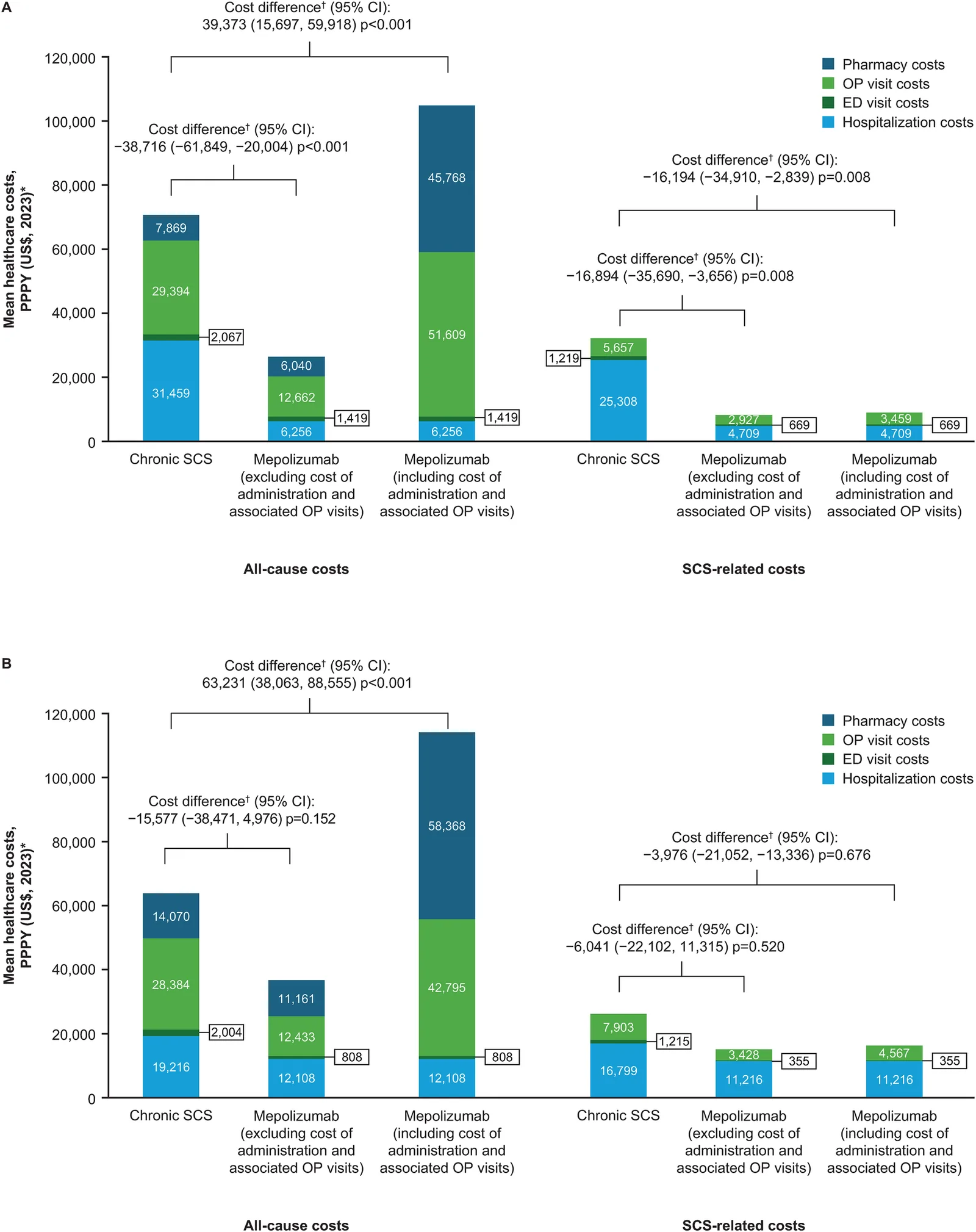
Healthcare costs including and excluding mepolizumab-related costs for **(A)** EGPA and **(B)** HES. *Costs were inflation-adjusted to $US 2023 using the U.S. Medical Care consumer price index from the U.S. Department of Labor Bureau of Labor Statistics; ^†^cost differences are reported for IPTW weighted cohorts. CI, confidence interval; ED, emergency department; EGPA, eosinophilic granulomatosis with polyangiitis; HES, hypereosinophilic syndrome; IPTW, inverse probability of treatment weighting; OP, outpatient; PPPY, per person per year; SCS, systemic corticosteroid.

When mepolizumab-related costs were excluded, patients with EGPA treated with mepolizumab vs. chronic SCS had lower all-cause total healthcare costs (cost difference [95% CI]: −$38,716 [−61,849, −20,004], *p* < 0.001), total medical costs, and pharmacy costs. When mepolizumab-related costs were included all-cause total healthcare costs were significantly higher for the mepolizumab cohort, compared with the chronic SCS cohort ($39,373 [15,697, 59,918], *p* < 0.001), due to higher pharmacy and outpatient visit costs associated with the acquisition and administration of mepolizumab. All-cause hospitalization costs were significantly lower in patients treated with mepolizumab compared with chronic SCS (Figure 4A).

In patients with EGPA, SCS-complication-related total medical costs were significantly lower for patients treated with mepolizumab vs. chronic SCS irrespective of whether mepolizumab-related costs were included or excluded (cost difference including mepolizumab-related costs [95% CI]: −$16,194 [−34,910, −2,839] *p* = 0.008; Figure 4A). The largest part of this difference was due to significant reductions in SCS-related hospitalization costs.

When mepolizumab-related costs were excluded, patients with HES treated with mepolizumab had significantly lower costs associated with outpatient visits compared with patients treated with chronic SCS (mean difference [95% CI]: −$10,424 [−20,967, −1,491], *p* = 0.016). When mepolizumab-related costs were included, all-cause total healthcare costs were significantly higher in the mepolizumab cohort compared with the chronic SCS cohort ($63,231 [38,063, 88,555], *p* < 0.001), due to higher pharmacy and outpatient visit costs associated with the acquisition and administration of mepolizumab. There was a numerical reduction in SCS complication-related total medical costs for mepolizumab users compared with chronic SCS users, when mepolizumab-related costs were excluded or included (Figure 4B).

### 3.7 Sensitivity analyses

The results of the dosage sensitivity analysis, in which patients in the mepolizumab user cohorts were eligible if they had ≥2 claims for mepolizumab 100 mg (the approved dose for indications other than HES and EGPA) or 300 mg within the landmark period (other eligibility criteria were unchanged), align with the overall results described for both patients with EGPA and HES, as described in Supplementary Results (Sensitivity analyses results). In brief, patients with EGPA in the mepolizumab 100 mg or 300 mg cohort compared with the chronic SCS cohort had a 36% (*p* < 0.001) lower rate of any SCS-related complication over the observation period compared with patients treated with chronic SCS. In patients with HES, mepolizumab users had a 67% (*p* = 0.004) lower rate of any acute complication relative to patients treated with chronic SCS.

Findings from the diagnosis sensitivity analysis, for which patients had to meet a more stringent eligibility criteria for both EGPA or HES diagnoses, align with the overall results described for both patients with EGPA and HES, as described in Supplementary Results (Sensitivity analysis results). Patients with EGPA treated with mepolizumab had a 55% (*p* < 0.001) lower rate of any SCS-related complication over the observation period relative to patients treated with chronic SCS. Similarly, patients with HES treated with mepolizumab had a 43% (*p* = 0.024) lower rate of any SCS-related complication over the observation period compared with patients treated with chronic SCS.

## 4 Discussion

This longitudinal, retrospective cohort study is the first to our knowledge to compare SCS-related complications, HRU, and associated costs between mepolizumab and chronic SCS treatment in patients with EGPA and HES in the US. It demonstrated that mepolizumab is associated with significantly lower rates of both acute and chronic SCS-related complications, as well as reduced rates of hospitalizations and outpatient visits in patients with EGPA and HES compared with chronic SCS use, and lower SCS-complication-related healthcare costs in patients with EGPA. These findings build on the known steroid-sparing effects of mepolizumab (22, 27, 28, 31–33), to offer insight into specific benefits not previously characterized nor quantified for patients with EGPA or HES. These findings also support the guidance from the American College of Rheumatology, European Alliance of Associations for Rheumatology, and the British Society for Rheumatology regarding use of steroid-sparing agents such as mepolizumab (9–11).

The consequences of SCS use, especially long-term therapy in patients with chronic conditions such as asthma and AAV have been well documented (15, 16, 19, 37). The use of the glucocorticoid toxicity index found that patients with AAV, which includes EGPA, experience significant levels of SCS-related complications due to the high levels of SCS exposure (19). Additionally, the odds of developing chronic conditions such as hypertension and reduced renal function were found to be significantly associated with SCS use in patients with AAV (38). Due to the chronic inflammatory nature of EGPA and HES, patients often require chronic SCS treatment to manage symptoms and can therefore experience a substantial treatment-related burden from SCS-related complications (14, 19). The importance of the use of steroid-sparing therapies is reflected by the current treatment guidelines, which emphasize the importance of using the minimal effective SCS dose alongside steroid-sparing therapies (9–12), and in patient perspectives (6, 39).

Mepolizumab treatment has been shown to reduce the need for SCS in both EGPA and HES. Over the 52-weeks of the Phase III MIRRA trial in patients with EGPA, cumulative SCS exposure was substantially reduced by an equivalent of prednisone 4.3 mg per patient per day in patients receiving mepolizumab vs. placebo and 18% vs. 2% of patients were able to discontinue SCS (22, 27). This effect improved over time as 31% of patients treated with mepolizumab discontinued SCS completely for ≥3 months during an open-label extension study (29). Mepolizumab treatment demonstrated similar SCS sparing effects in patients with HES, as 28% of patients treated with mepolizumab during a 20-week open-label extension study were able to reduce SCS use by at least 50%, with mean daily OCS doses reducing from 11.2 to 8.9 mg (32). The steroid-sparing benefits of mepolizumab were further evidenced in the MANDARA non-inferiority trial, where treatment with either mepolizumab or benralizumab, an anti-IL 5 receptor biologic, allowed for substantial reductions in SCS use in patients with EGPA (40).

The present study demonstrates that these steroid-sparing effects may translate to significant real-world reduction in SCS-related toxicities, including infections and acute cardiovascular complications including myocardial infarction in both patient populations. The rate of chronic cardiovascular conditions such as hypertension, and metabolic and endocrine conditions commonly associated with SCS use, were also significantly reduced in both cohorts (16). However, this change in reported chronic complications was detected only over the relatively short 6-month treatment effect period of this study, and longer-term studies would be required to identify if more pronounced effects emerge over time. In addition, the results of the dosage sensitivity analysis, which included patients administered mepolizumab 100 mg or 300 mg, reflecting real-world prescribing practices, confirmed benefits in reduction in SCS-associated complications regardless of initial dosage. Similar findings have been demonstrated in patients with severe asthma, where patients treated with mepolizumab had lower rates of both chronic and acute SCS-related complications than patients treated with chronic SCS (6–12 or ≥12 mg/day) (41). Importantly, these findings provide tangible support to the notion that reduction of steroid-related toxicity, and not simply reduction in steroid use, should be considered for inclusion in updated treatment goals for both HES and EGPA. Results also support current treatment guidance regarding the use of biologics for remission maintenance (10), and align with recent demonstrations that with biologic therapy SCS-free remission can be a feasible long-term treatment goal for patients with EGPA (42).

Additionally, mepolizumab use was associated with decreased HRU and offered some cost savings compared with chronic SCS use in the management of EGPA and HES. The burden of both diseases on patients and healthcare systems is known to be high, with a recent comparison demonstrating that patients with EGPA utilized significantly more healthcare services such as hospitalizations, ED visits, and outpatient visits than patients with asthma (4, 5, 14, 43, 44). The findings from the present study show that mepolizumab use was linked to reduced all-cause and SCS-related hospitalizations and reduced the need for ED visits related to SCS-complications in EGPA and HES. When mepolizumab-associated costs were accounted for, total healthcare costs were higher for the mepolizumab cohort compared with the chronic-SCS cohort for patients with EGPA and HES, with pharmacy and outpatient visits costs associated with mepolizumab administration being the main driver of higher costs. However, these costs were partly offset by the reductions in SCS-complication related costs, which were $16,194 lower in patients with EGPA receiving mepolizumab compared with chronic-SCS. Patients with HES experienced numerically lower total SCS-related costs with mepolizumab treatment vs. chronic SCS. Hospitalization costs remained lower for both patients with EGPA and HES treated with mepolizumab compared with chronic SCS, irrespective of whether mepolizumab-associated costs were included or excluded. The financial burden of both diseases has been highlighted by patients and caregivers, who may struggle to afford treatment and appointments (45); these findings may allow patients and prescribers to be better informed on the economic impact of SCS use.

Taken together, these reductions in SCS-related complications, along with reduced HRU and costs suggest that the use of mepolizumab is associated with a lower burden of disease on both patients and healthcare systems. This may in turn have a positive impact on patient quality of life, further emphasizing the importance of steroid-sparing agents in the management of these diseases. Patients with EGPA and HES are affected by numerous symptoms including shortness of breath and fatigue, impacting sleep, ability to exercise, engage in social, and work, in turn negatively impacts patient quality of life (6, 46–48). Mepolizumab has been demonstrated to reduce flares and symptom burden, and to positively impact ability to work and overall health-related quality of life in patients with HES (23, 47, 49), and is associated with reduced disturbance from symptoms and improved health-related quality of life in patients with EGPA (48).

Limitations of the present study should be considered when interpreting the results, many of which are inherent to retrospective claims analyses. Firstly, the use of administrative claims data collected for payment rather than for research purposes for assessment of the primary outcomes of this study may leave the data vulnerable to coding inaccuracies. The presence of a diagnosis code in patient records may not reflect the true presence of the disease due to possible inaccuracies in coding or diagnosis codes being included as a rule-out criterion. To mitigate this, a more rigorous set of diagnostic criteria were used in the diagnosis sensitivity analysis. Findings from the diagnosis sensitivity analysis were consistent with the main analysis, which supports the reliability of using ICD-10-CM codes to identify patients with EGPA and HES in administrative claims data and strengthens the generalizability of the findings. In addition, pharmacy claims, while considered a good measure of medication use, do not guarantee patient compliance with the prescribed/dispensed medication, and therefore may inaccurately reflect medication utilization.

Moreover, complications associated with SCS use may overlap with clinical manifestations of EGPA and HES, leading to inaccuracies in reporting of SCS-related complications, HRU, and costs. Although this overlap introduces potential for misclassification, the impact of this on the outcomes was minimized by the inclusion of baseline SCS-related complications in the propensity score model. Any such misclassification is therefore expected to affect both groups similarly during follow-up. By adjusting for baseline differences, the IPTW approach reduces potential for confounding to impact the results, although not all confounders can be adjusted for with this study design. Lastly, the follow-up duration available within this dataset was relatively limited, which constrains the ability to fully characterize the long-term, chronic nature of SCS-related complications that may evolve over extended periods.

We acknowledge that selecting the chronic SCS cohort as a comparator may introduce confounding and bias, due to potential differences in disease severity, treatment history, and access to biologic therapies such as mepolizumab. However, we consider that this is representative of real-world patients with EGPA and HES with ongoing high disease burden who require continuous or frequent steroid use to control disease. This is reflected by the high cumulative baseline SCS dosage seen in both the mepolizumab and SCS cohorts in both HES and EGPA (Table 1). This deliberate selection allows us to initially evaluate whether the relatively novel endpoint of reductions in steroid-related complications can be demonstrated, and to what extent. By focusing on this population, the likelihood of detecting meaningful differences was maximized while ensuring the findings are applicable to real-world patients most in need of improved therapeutic strategies.

To our knowledge, this is the only real-world analysis to date that has sought to understand the impact of the steroid-sparing benefits of mepolizumab in HES and EGPA. There remains limited data on SCS-related complications in these rare diseases and future studies could build on these findings via different methodologies including patient chart review or within the context of prospective, randomized, controlled studies for patients with EGPA or HES. Additionally, such studies would benefit from the use of a validated instrument to quantify SCS-related toxicity such as the glucocorticoid toxicity index, which has been shown to effectively demonstrate SCS toxicity in conditions such as EGPA (19, 50). This may be a useful tool for identifying patients who may be at increased risk of SCS-related complications and achieve lasting benefits from steroid-sparing treatment.

## 5 Conclusions

Results from this large US administrative claims study showed that mepolizumab is associated with statistically significant reductions in SCS-related complications, including both acute and chronic complications. Additionally, associated reductions in HRU and related costs support the clinical and economic benefits of steroid-sparing agents such as mepolizumab among patients with EGPA and HES. Consequently, mepolizumab may help patients with these diseases, who face high disease and treatment burdens, reach guideline-based goals of achieving disease control while reducing the risk of SCS-related complications, which could positively impact quality of life.

## Data Availability

The original contributions presented in the study are included in the article/Supplementary Material, further inquiries can be directed to the corresponding author/s.
