# Supplementary material for: Mepolizumab reduces systemic corticosteroid-related toxicities and healthcare burden in patients with eosinophilic granulomatosis with polyangiitis and hypereosinophilic syndrome: a retrospective database study
Source: Front Allergy. 2026 Aug 12;7:1843149. doi: 10.3389/falgy.2026.1843149 (PMC13509139; doi:10.3389/falgy.2026.1843149)
Supplement: Supplementary file 1 [file Datasheet1.docx]

**Supplementary Materials**

**Contents**

[**Plain Language Summary	2**](#_Toc230009537)

[**Supplementary methods	3**](#_Toc230009538)

[Propensity score covariates	3](#_Toc230009539)

[Sensitivity analyses methods	4](#_Toc230009540)

[**Supplementary results	4**](#_Toc230009541)

[Sensitivity analyses results	4](#_Toc230009542)

[**Supplementary tables and figures	6**](#_Toc230009543)

[Supplementary Figure 1. Identification (green) and exclusion (yellow) criteria for patients with EGPA treated with mepolizumab or chronic SCS	6](#_Toc230009544)

[Supplementary Figure 2. Identification (green) and exclusion (yellow) criteria for patients with HES treated with mepolizumab or chronic SCS.	7](#_Toc230009545)

[Supplementary Table 1. SCS-related complications*	8](#_Toc230009546)

[Supplementary Table 2. Baseline HRU and healthcare costs in patients with EGPA and HES (IPTW weighted cohorts)	9](#_Toc230009549)

**Plain Language Summary**

Eosinophilic granulomatosis with polyangiitis (known as EGPA) and hypereosinophilic syndrome (known as HES) are rare diseases where too many eosinophils (cells in the immune system) damage organs. These diseases are commonly treated with steroids tablets or injections. Steroids can cause serious side effects, such as weak bones and bruising. Mepolizumab is a type of injected targeted medicine (a biologic) that reduces eosinophil levels and helps patients use less steroids.

We looked at insurance claims from patients with EGPA and HES for a period of up to 12 months, to see whether taking mepolizumab could lead to fewer steroid side effects and lower the need for medical care and its costs. Patients with EGPA and HES who took mepolizumab were compared with patients who took regular steroids and no mepolizumab. Patients had used mepolizumab or steroids for 6 months before we looked at their insurance claims.

The study included 578 patients with EGPA and 272 patients with HES. We found that patients with EGPA who took mepolizumab had 39% fewer steroid side effects and lower medical costs (excluding the cost of mepolizumab) than those who only took steroids. For patients with HES, side effects from steroids were 41% less common in those who took mepolizumab. Patients with EGPA and HES taking mepolizumab also had fewer emergency department visits and hospital stays due to steroid side effects.

This study builds on previous studies that showed that mepolizumab can help people with EGPA or HES to reduce the amount of steroids needed to manage their disease. This study showed that patients treated with mepolizumab had fewer side effects caused by steroids. It also helps reduce the need for emergency medical care and hospital stays.

**Supplementary methods**

Propensity score covariates

Covariates included in the propensity score used to generate IPTW weights were:

- Age at index date
- Sex
- Index year
- US geographic region
- Insurance plan type
- Physician specialty
- Quan-Charlson Comorbidity Index (CCI)
- History of any controller medications, rescue medications and immunosuppressants during baseline
- EGPA relapse
- Asthma during eligibility period
- Number of asthma-related exacerbations (inpatient and outpatient/ED)
- Number of nasal polyp-related exacerbations
- All-cause HRU during baseline (inpatient visits, outpatient visits, and ED visits)
- All-cause healthcare costs during baseline (total medical costs and pharmacy costs)
- Cumulative baseline SCS dosage
- Any acute SCS-related complications (infections, gastrointestinal, and cardiovascular)
- Any chronic SCS-related complications (cardiovascular, metabolic and endocrine, central nervous system, bone- and muscle-related, ophthalmologic, and hematologic)
- Baseline Elixhauser comorbidities (hypertension, rheumatoid arthritis/collagen vascular disease, fluid and electrolyte disorders, diabetes, congestive heart failure, and valvular disease)

Sensitivity analyses methods

Two sensitivity analyses were performed to test the robustness of study results. A dosage sensitivity analysis was conducted, replicating the study outcomes among patients with EGPA and HES treated with mepolizumab 100 mg (the approved dose for indications other than HES and EGPA) or 300 mg versus chronic SCS.

A diagnosis sensitivity analysis was conducted to validate the diagnostic criteria used in the main analysis. Patients with EGPA included in the diagnosis sensitivity analysis required ≥2 diagnoses of EGPA, ≥1 diagnosis of asthma, and ≥1 diagnosis of any of the EGPA-related conditions (i.e., vasculitis, neuropathy, nasal polyps, cardiomyopathy, glomerulonephritis, alveolar hemorrhage, palpable purpura) during the baseline period. Patients with HES included in the diagnosis sensitivity analysis required ≥2 diagnoses of HES, and no evidence of parasitic helminth infections, human immunodeficiency virus, drug rash with eosinophilia and systemic symptoms, myeloproliferative disorders, or leukemias during the baseline period.

**Supplementary results**

Sensitivity analyses results

*Dosage sensitivity analysis*

A mepolizumab dosage sensitivity analysis was conducted in which patients in the mepolizumab user cohorts were eligible if they had ≥2 claims for mepolizumab 100 mg or 300 mg within the landmark period (other eligibility criteria were unchanged). This identified 369 patients with EGPA and 234 patients with HES treated with mepolizumab 100 mg or 300 mg. The chronic SCS cohorts were unchanged from the main analysis. Results in this expanded population receiving either mepolizumab 100 mg or 300 mg were consistent with the mepolizumab 300 mg data. Patients with EGPA in the mepolizumab 100 mg or 300 mg cohort compared with the chronic SCS cohort had a significantly lower rate of any SCS-related complication over the observation period compared with patients treated with chronic SCS (rate ratio [95% CI]: 0.64 [0.52, 0.79], p<0.001). Additionally, patients treated with mepolizumab had significantly lower rates of all-cause hospitalizations, and SCS-complication-related hospitalizations, ED, and outpatient visits relative to patients treated with chronic SCS. In patients with HES, mepolizumab users had a 67% lower rate of any acute complication relative to patients treated with chronic SCS users (0.33 [0.15, 0.71], p=0.004), with significantly lower rates of chronic bone- and muscle-related and chronic hematologic complications. Rates of all-cause hospitalizations and outpatient visits, and SCS-complication-related hospitalizations were also significantly lower in mepolizumab users relative to chronic SCS users.

*Diagnosis sensitivity analysis*

After applying additional diagnostic criteria to identify patients with EGPA and HES, 206 patients with EGPA (135 mepolizumab users, and 71 chronic SCS users), and 149 patients with HES (107 mepolizumab users, 42 chronic SCS users) were included. Findings from the diagnosis sensitivity analysis align with the overall results described for both patients with EGPA and HES. Patients with EGPA treated with mepolizumab had a significantly lower rate of any SCS-related complication over the observation period relative to patients treated with chronic SCS (rate ratio [95% CI]: 0.45 [0.32, 0.63], p<0.001). Mepolizumab users with EGPA had significantly lower rates of all-cause hospitalizations and of SCS complication-related hospitalizations, ED, and outpatient visits relative to the chronic SCS cohort. Similarly, patients with HES treated with mepolizumab had a significantly lower rate of any SCS-related complication over the observation period compared with patients treated with chronic SCS (0.57 [0.35, 0.93], p=0.024). Patients with HES treated with mepolizumab had significantly lower rates of all-cause hospitalizations and outpatient visits, and SCS-complication-related hospitalizations compared with chronic SCS use.

**Supplementary tables and figures**

**Supplementary Figure 1. Identification (green) and exclusion (yellow) criteria for patients with EGPA treated with mepolizumab or chronic SCS**

*EGPA was identified using ICD-10-CM diagnosis code M30.1.;^†^all patients were required to have ≥6 months of continuous use of oral or parenteral SCS after the index date in the prior step, and therefore no patients were excluded at this step; ^‡^eligibility period defined as the period of eligible insurance coverage encompassing the baseline, landmark, and observation periods.

EGPA, eosinophilic granulomatosis with polyangiitis; FDA, US Food and Drug Administration; ICD-10-CM: International Classification of Diseases, Tenth Revision, Clinical Modification; SCS, systemic corticosteroid.

**Supplementary Figure 2. Identification (green) and exclusion (yellow) criteria for patients with HES treated with mepolizumab or chronic SCS.**

*HES was identified using ICD-10-CM diagnosis code D72.11x.;^†^all patients were required to have ≥6 months of continuous use of oral or parenteral SCS after the index date in the prior step, and therefore no patients were excluded at this step; ^‡^eligibility period defined as the period of eligible insurance coverage encompassing the baseline, landmark, and observation periods.

HES, hypereosinophilic syndrome; US FDA, Food and Drug Administration; ICD-10-CM, International Classification of Diseases, Tenth Revision, Clinical Modification; SCS, systemic corticosteroid.

**Supplementary Table 1. SCS-related complications***

| **Category** | **Acute/Chronic** | **Condition** |
| --- | --- | --- |
| **Gastrointestinal** | Acute | Nausea/Vomiting |
|  |  | Gastrointestinal bleeds/Ulcers |
|  |  | Dyspepsia |
|  |  | Abdominal distension |
|  |  | Acute pancreatitis |
|  | Chronic | Chronic gastrointestinal bleeds/Ulcers |
| **Infections** | Acute | Fungal infection |
|  |  | Pneumonia |
|  |  | Tuberculosis |
|  |  | Urinary tract infection |
|  |  | Varicella infection |
|  |  | Sepsis |
| **Bone and muscle** | Acute | Bursitis |
|  | Chronic | Fractures |
|  |  | Avascular necrosis |
|  |  | Muscle weakness |
|  |  | Loss of muscle mass |
|  |  | Osteoporosis |
|  |  | Back pain |
|  |  | Myopathy |
| **Cardiovascular** | Acute | Myocardial infarction |
|  | Chronic | Congestive heart failure |
|  |  | Atrial fibrillation/Flutter |
|  |  | Hypertension |
|  |  | Stroke |
| **Hematologic** | Chronic | Epistaxis |
|  |  | Leukocytosis |
| **Metabolic and endocrine** | Chronic | Cushing Syndrome |
|  |  | Obesity |
|  |  | Diabetes mellitus |
|  |  | Metabolic syndrome |
|  |  | Hyperglycemia |
|  |  | Dyslipidemia |
|  |  | Drug-induced adrenocortical insufficiency |
| **Ophthalmologic** | Chronic | Cataracts |
|  |  | Glaucoma |
| **Central nervous system** | Chronic | Bipolar disorder |
|  |  | Depression |
|  |  | Sleep disturbances |
|  |  | Steroid psychosis |
|  |  | Migraines |
|  |  | Akathisia |
| **Dermatologic** | Chronic | Acne |
|  |  | Erythema |
|  |  | Hirsutism |

*SCS-related complications were identified for inclusion based on having been reported as associated with SCS use in previous literature (18, 34, 35)

SCS, systemic corticosteroid.

**Supplementary Table 2. Baseline HRU and healthcare costs in patients with EGPA and HES (IPTW weighted cohorts)**

|  | **EGPA** | | | **HES** | | |
| --- | --- | --- | --- | --- | --- | --- |
| **Characteristic** | **Mepolizumab cohort** | **Chronic SCS cohort** | **Std. diff. (%)** | **Mepolizumab cohort** | **Chronic SCS cohort** | **Std. diff. (%)** |
|  | **N=305** | **N=273** |  | **N=161** | **N=111** |  |
| **All-cause HRU during baseline, mean (SD) [median]** |  |  |  |  |  |  |
| IP visits | 0.5 (1.1) [0.0] | 0.6 (1.1) [0.0] | 10.90 | 0.7 (1.0) [0.0] | 0.8 (1.2) [0.0] | 10.60 |
| ED visits | 1.4 (3.6) [0.0] | 1.6 (4.2) [1.0] | 5.30 | 1.4 (1.9) [1.0] | 1.5 (1.7) [1.0] | 5.70 |
| OP visits | 32.5 (20.0) [27.0] | 32.6 (24.7) [28.0] | 0.20 | 36.1 (26.5) [28.0] | 36.1 (45.3) [27.0] | 0.00 |
| **All-cause healthcare costs during baseline^*^, USD 2023, mean (SD) [median]** |  |  |  |  |  |  |
| Total healthcare (medical + pharmacy) costs | 46,418.9 (85,547.4) [30,109.2] | 49,622.4 (79,325.9) [23,644.8] | 3.90 | 52,929.4 (63,574.6) [32,276.5] | 56,660.5 (72,109.3) [30,530.4] | 5.50 |
| Total medical costs | 38,059.2 (84,562.0) [20,376.2] | 41,816.1 (76,825.5) [15,321.5] | 4.70 | 43,170.2 (60,112.6) [28,390.8] | 48,879.4 (67,458.9) [23,903.6] | 8.90 |
| IP visit costs | 17,550.6 (73,782.7) [0.0] | 22,959.6 (70,916.7) [0.0] | 7.50 | 22,763.6 (52,281.8) [0.0] | 28,505.2 (53,060.3) [0.0] | 10.90 |
| ED visit costs | 1468.9 (3,821.3) [0.0] | 1612.0 (3,162.7) [145.5] | 4.10 | 1889.6 (4,065.8) [399.8] | 2527.8 (4,194.9) [684.9] | 15.40 |
| OP visit costs | 19,039.7 (23,314.8) [10,301.7] | 17,244.6 (21,752.0) [9,889.9] | 8.00 | 18,516.9 (20,515.9) [11,412.4] | 17,846.4 (29,452.4) [8,982.7] | 2.60 |
| Pharmacy costs | 8394.5 (12,025.4) [4,036.1] | 7806.2 (15,494.5) [4,226.7] | 4.20 | 9793.5 (20,973.9) [3,360.8] | 7781.1 (16,623.9) [1,840.5] | 10.60 |

*Costs were inflation-adjusted to $US 2023 using the U.S. Medical Care consumer price index from the U.S. Department of Labor Bureau of Labor Statistics

EGPA, eosinophilic granulomatosis with polyangiitis; HES, hypereosinophilic syndrome; ED, emergency department; HRU, health resource utilization; IP, inpatient; OP, outpatient; IPTW, inverse probability of treatment weighting; SCS, systemic corticosteroids; SD, standard deviation; Std. diff., standardized difference; US, United States; USD, United States dollar.

**Supplementary Table 3. SCS use during baseline and observation among mepolizumab vs chronic SCS cohorts in patients with EGPA and HES**

|  | **EGPA** | | **HES** | |
| --- | --- | --- | --- | --- |
| **Systemic corticosteroid use** | **Mepolizumab cohort** | **Chronic SCS cohort** | **Mepolizumab cohort** | **Chronic SCS cohort** |
|  | **N=305** | **N=273** | **N=161** | **N=111** |
| **Baseline period** |  |  |  |  |
| Cumulative SCS dosage* during baseline, mg, mean (SD) [median] | 1,843.2 (1,511.2) [1,590.0] | 1,846.7 (1,443.4) [1,620.0] | 1,360.8 (1,223.7) [1,100.0] | 1,392.2 (1,459.2) [825.0] |
| **Observation period** |  |  |  |  |
| Mean number of SCS dispensings, PPPY, mean (SD) [median] | 11.0 (18.6) [6.2] | 26.2 (47.7) [18.0] | 9.6 (21.4) [3.0] | 25.9 (33.1) [17.3] |
| Patients with ≥1 SCS dispensing, n (%) | 266 (87.3) | 269 (98.5) | 120 (74.4) | 111 (100.0) |
| Mean number of SCS dispensings, PPPY, mean (SD) [median] | 12.6 (19.5) [8.0] | 26.6 (47.7) [18.0] | 12.9 (24.5) [5.0] | 25.9 (33.1) [17.3] |
| Daily dose per SCS dispensing^†^, mg, mean (SD) [median] | 18.4 (26.1) [10.0] | 10.0 (3.8) [9.1] | 20.0 (33.9) [10.6] | 11.4 (4.5) [10.0] |
| Cumulative SCS dosage* during observation, mg, mean (SD) [median] | 225.1 (240.7) [145.0] | 473.7 (190.1) [484.0] | 167.4 (194.3) [68.0] | 387.1 (161.5) [360.0] |
| Patients with no SCS dispensing, n (%) | 39 (12.7) | 4 (1.5) | 41 (25.6) | 0 (0.0) |

*Cumulative dose was defined as the sum of the quantity × prednisone equivalent strength over the given period in milligrams in patients with ≥1 SCS dispensing during observation. For medical claims with missing quantity information, quantity was imputed as 1; ^†^the average daily dose of SCS per dispensing was calculated as number of tablets × prednisone equivalent strength/number of days supplied in patients with ≥1 SCS dispensing during observation. For medical claims with missing quantity information, quantity was imputed as 1.

EGPA, eosinophilic granulomatosis with polyangiitis; HES, hypereosinophilic syndrome; PPPY, per person per year; SCS, systemic corticosteroid; SD, standard deviation.
